# Molecular Systems Biology of Neurodevelopmental Disorders, Rett Syndrome as an Archetype

**DOI:** 10.3389/fnint.2019.00030

**Published:** 2019-07-17

**Authors:** Victor Faundez, Meghan Wynne, Amanda Crocker, Daniel Tarquinio

**Affiliations:** ^1^Department of Cell Biology, Emory University, Atlanta, GA, United States; ^2^Program in Neuroscience, Middlebury College, Middlebury, VT, United States; ^3^Rare Neurological Diseases (Private Research Institution), Atlanta, GA, United States

**Keywords:** Rett, autism, biomarker, clinical trials, genealogical proteomics, precision medicine

## Abstract

Neurodevelopmental disorders represent a challenging biological and medical problem due to their genetic and phenotypic complexity. In many cases, we lack the comprehensive understanding of disease mechanisms necessary for targeted therapeutic development. One key component that could improve both mechanistic understanding and clinical trial design is reliable molecular biomarkers. Presently, no objective biological markers exist to evaluate most neurodevelopmental disorders. Here, we discuss how systems biology and “omic” approaches can address the mechanistic and biomarker limitations in these afflictions. We present heuristic principles for testing the potential of systems biology to identify mechanisms and biomarkers of disease in the example of Rett syndrome, a neurodevelopmental disorder caused by a well-defined monogenic defect in methyl-CpG-binding protein 2 (MECP2). We propose that such an approach can not only aid in monitoring clinical disease severity but also provide a measure of target engagement in clinical trials. By deepening our understanding of the “big picture” of systems biology, this approach could even help generate hypotheses for drug development programs, hopefully resulting in new treatments for these devastating conditions.

## Introduction

Rett syndrome is a devastating neurodevelopmental disorder caused by mutations in a gene responsible for both activating and repressing gene transcription: methyl CpG binding protein 2 gene (*MECP2*; Amir et al., 1999). Rett syndrome is an X linked disease that predominantly affects females (prevalence approximately 1:10,000 females; Leonard et al., 1997; Bienvenu et al., 2006; Wong and Li, 2007). Through the process of lyonization (X-chromosome inactivation), patient tissues become mosaic for *MECP2*, as both normal and mutated versions of *MECP2* are expressed. The ratio of mutant to non-mutant protein in mosaic tissue is in part responsible for determining the severity of the disorder in the individual (Amir et al., 2000). Although apparently normal in early infancy, children with Rett syndrome fail to achieve milestones in late infancy, then undergo a period of regression of language and hand use, followed by emergence of pervasive repetitive hand movements known as stereotypies. The regression period is often associated with social withdrawal, and the disorder has been classified in the past as part of the autism spectrum (Percy, 2011). However, after children with Rett syndrome emerge from the regression period, they enter a phase of stability, often with subtle developmental gains or losses, but almost never regain meaningful verbal language or hand use (Downs et al., 2010). They require constant care, often living into their 5th or 6th decade with waxing and waning periodic medical and neurological comorbidities, including epilepsy, periodic breathing disorder, disturbances of mood and behavior, pervasive growth failure, scoliosis, movement disorder, various sleep disorders, osteopenia, abnormal pubertal development, electrocardiograms with prolonged cardiac QT interval, and numerous gastrointestinal disorders (Glaze et al., 1987; FitzGerald et al., 1990; Ellaway et al., 1999; Motil et al., 2012; Tarquinio et al., 2012, 2015, 2017, 2018; Cuddapah et al., 2014; Killian et al., 2014; Jefferson et al., 2016).

Few neurodevelopmental disorders appear as amenable to targeted treatment as Rett syndrome based on preclinical evidence (Pozzo-Miller et al., 2015; Katz et al., 2016). Neurons in both individuals with Rett syndrome and mice with *Mecp2* mutations undergo normal migration but suggest developmental arrest of synaptic connections (Armstrong, 2005; Chapleau et al., 2009). No evidence of degeneration exists, and several studies have demonstrated rescue of neuropathological abnormalities in mouse models, even in adult animals (Guy et al., 2007; Robinson et al., 2012; Garg et al., 2013). Despite this evidence, human trials have failed to produce clinically meaningful change (Katz et al., 2016). On closer examination, although the preclinical evidence supporting therapeutic strategies appears strong, these disappointing trial results may stem from faulty assumptions about how these results would translate into humans. These assumptions can be divided into two broad categories: (1) mechanistic assumptions about MECP2 function; and (2) efficacy assumptions regarding how specific outcomes seen in a murine model would actually present in a human.

Molecular strategies using “omic” approaches can help to inform both the mechanisms of MeCP2 dysfunction and the pathophysiological changes we would expect to see in humans if these dysregulated mechanisms were put right. These strategies help to fill in gaps in our understanding of how dysregulated transcription of the targets of MeCP2 can result in such a protean disorder as Rett syndrome. Moreover, the findings of a comprehensive “omic” approach could result in biomarkers at various levels downstream of MeCP2. Optimally, this would result in molecular biomarkers to differentiate which populations of patients will respond best to a specific treatment, and at what developmental stage, to optimize dosing of treatment. Such biomarkers would also serve as a surrogate outcome measure of improvement in the core characteristics of disease and associated comorbidities. The omics approach accounts for the role fundamental biological components play in disease, and an omics-based biomarkers discovery program would allow for translation from basic molecular mechanisms to clinically meaningful surrogate outcome measures. A deep understanding of “omic”-based molecular phenotypes in Rett syndrome could provide a portfolio of biomarkers suitable for many drug development and clinical trial approaches.

In an effort to both improve outcome measures and develop biomarkers for Rett syndrome, the multi-center Rett syndrome Outcome Measures and Biomarker Development program^1^ was established. Over the past 2 years, the program has collected data on a host of caregiver-reported, clinician-reported, and performance outcome measures in Rett syndrome subjects, and also tested a number of approaches to biomarker data collection, ranging from biometric recordings of physiological function (ECG, induction plethysmography, galvanic skin response, accelerometer and gyroscope recording of movement) to sampling tissue. This review focuses on one of the most promising approaches we have investigated, that of global interrogation of tissue protein expression.

During its inception, the principal investigators considered a number of targeted biomarkers in serum, cerebrospinal fluid (CSF), and other tissues. These included hormones such as leptin, ghrelin, and adiponectin (Blardi et al., 2009; Hara et al., 2011), and cortisol levels (Echenne et al., 1991). We also considered physiological markers such as skin temperature (Symons et al., 2015), and both eye tracking and pupillometry (Farzin et al., 2011; Rose et al., 2013). Since our initial review, other targeted markers have received attention, including immune and enzymatic markers as well as neurophysiological tests such as auditory and visual evoked potentials (Papini et al., 2014; LeBlanc et al., 2015; Hayek et al., 2017; Key et al., 2019). Ultimately, since all of these biomarkers are far downstream to the regulatory effects exerted by the MeCP2 protein, we opted to focus on a minimally biased global approach to measure the effects of dysregulation due to loss of function in *MECP2*.

We have collected skin biopsies and whole blood on approximately two dozen families (often as trios with parents and affected child) and banked these tissues for testing of “omic” biomarkers. We are also currently in the process of evaluating the results of multi-tissue omics in the *Mecp2* null male mouse to evaluate the degree to which translational assumptions from the animal model to the human hold true. The focus on male mice, as a first step, stems from the fact that most published research in Rett mouse models has been carried out in males. This approach has been embraced in an effort to minimize experimental noise introduced by brain X chromosome mosaicism in female Rett models (Braunschweig et al., 2004; Chahrour and Zoghbi, 2007; Renthal et al., 2018). However, it is clear that studies in the male model of MeCP2 loss of function must be validated in female mice to rigorously validate the potential of these biomarkers for translation into the human disease.

## What Is the Systems Biology and Multi-“Omic” Approach, and Why Is It Relevant to Neurodevelopmental Disorders?

Neurodevelopmental disorders are profoundly complex. The hypothesis that they can be understood based on reducing them to their component parts is attractive, but not likely to be true. No disorders illustrate this case more clearly than the autism spectrum disorders, now recognized collectively as a common neurodevelopmental disability (Xu et al., 2018). Complex behavioral disorders involving multiple components of an intricate network warrant a complex explanation. Thus, the prospect that autism can be reduced to understanding the molecular biology of a single gene and protein product, so called “naïve reductionism,” is untenable (Bloom, 2001; Strange, 2005). The list of autism “risk” genes, currently over 1,000, grows each year, and, due to the multi-dimensional nature of the disorder, one would be led to believe that no unifying “cause” of autism could exist (Ayhan and Konopka, 2019)^2^.

Although a unifying explanation for such a complex disorder may seem far-fetched, examples of monogenetic disorders associated with autism, such as Rett syndrome, do exist. In these monogenetic disorders, perturbation of a single gene producing a single protein product causes complex neurodevelopmental disorders with a host of systemic comorbidities and striking heterogeneity. Because a deep understanding of these examples could prove seminal for this common disease, researchers have designed monogenic knockout animal models of these rare diseases and sought to understand neurodevelopmental disorders like autism from the base up (Sztainberg and Zoghbi, 2016). In the case of specific examples of syndromic autism, the explanation for how a single gene mutation can result in such a complex neurodevelopmental disorder often lies in the complex function of the protein product of the mutated gene; in Rett syndrome, MeCP2 regulates the transcription of a host of genes yet to be identified, which may number over 1,000 (Horvath and Monteggia, 2017).

While models of syndromic autism created to understand non-syndromic autism spectrum disorder, such as the mouse models of Rett syndrome and Fragile X syndrome, have reasonable construct validity and face validity, predictive validity, the ability to translate improvements in the animal to improvements in the human, has been a harder target to hit. A number of pathways amenable to human translation have been identified, and clinical trials have examined the effects of intervention in these pathways downstream of the dysfunctional protein. In these clinical trials, we expected that restoration of systems dysregulated by the causative gene would result in meaningful clinical improvement in humans. However, to date, results of these approaches have been disappointing in terms of clinical outcome measures.

### A Brief History of Clinical Investigations and Therapeutic Trials in Rett Syndrome

Historically, Rett syndrome was the first pervasive developmental disorder with an identified monogenetic cause (Neul and Zoghbi, 2004). Much can be understood about neurodevelopmental disorders in general by deepening our understanding of this prototypical disorder. To understand why the omics approach can be a useful addition to the drug development process for neurodevelopmental disorders, it helps to understand the approach to molecular investigation and clinical trials. As an illustrative example, we will discuss the history of these issues in Rett syndrome.

#### The Rett Pathological Phenotype

The search for viable therapeutic targets in Rett syndrome began with neuropathology. The brain of Rett syndrome patients is globally abnormal, with brain weight in all age groups reduced to 60%–88% of expected weight (Jellinger et al., 1988). Structural changes include reduced volume of frontal cortex and deep nuclei; as in Parkinson disease, the substantia nigra exhibits reduced pigmentation (Jellinger, 2003). Notably, the overall appearance of the brain is normal; however, the brain is smaller, and the neuropil is denser. Neurons are both smaller and more tightly packed, and dendrites are shorter with less mature arborization (Armstrong, 1997). Overall, the neuropathology indicates developmental arrest rather than degeneration of synaptic connections (Kaufmann et al., 2005). Because Rett syndrome was historically considered as a progressive disease, with passage to a “late motor degeneration,” researchers expected to find evidence of degeneration. The fact that the pathology is not consistent with the clinical decline originally attributed to patients with the disorder has led to a rethinking of the degenerative aspect of Rett syndrome (Bauman et al., 1995). Now most experts consider the normal neuronal migration, involvement of multiple neurotransmitter systems, and immature dendrites as suggestive of developmental arrest rather than neurodegeneration, and the period of arrest correlates with development in the third trimester or during early infancy (Armstrong, 2002). Together, these findings of stable developmental arrest hold promise for the premise of establishing a disease-modifying treatment.

#### Unraveling *MECP2* Dysfunction and Cellular Phenotype

Experimental models of Rett syndrome have helped to elucidate the neuropathological phenotype seen in humans. In murine models with mutations in *Mecp2*, both in cases of deficient or absent protein, early development is normal, after which synapses fail to mature and synaptic reorganization is deficient (Boggio et al., 2010). Recently, the structure of *MECP2* was examined and the contribution of mutations to its structural destabilization elucidated, yet the molecular mechanisms linking abnormal MeCP2 function and Rett syndrome remain largely unclear (Spiga et al., 2019). There is a wide gap of molecular knowledge between the genotype and the phenotype, which we refer to here as the mesoscale gap, encompassing how cells, tissues and organs behave in the presence of a *MECP2* mutation. A number of general explanations have been proposed to explain the mesoscale gap., Evidence supports the notions that calcium-dependent activation is abnormal in response to synaptic stimulation, and that the loss of MeCP2’s epigenetic function disrupts synaptic reorganization (Chen et al., 2003). The concept of “synaptopathy” has been related to many of the clinical features present in Rett syndrome patients. Indeed, long-term potentiation is normal in early life in Mecp2 deficient mice; however, when they become symptomatic, long-term potentiation becomes abnormal, consistent with the clinical regression of language and hand use seen in patients (Weng et al., 2011). Along with decreased Mecp2 levels, the post-synaptic protein PSD-95 is decreased, and both excitatory and inhibitory signaling are abnormal (Chao et al., 2007).

The protein MeCP2 is primarily an epigenetic protein, responsible for both repression and induction of gene transcription, as well as regulation of chromatin organization (Lyst and Bird, 2015). While MeCP2 is primarily expressed in brain tissue, the protein can be found expressed in all tissues (Kaddoum et al., 2013). When MeCP2 is either absent or functions abnormally, this results in immature neurons. Several mechanisms for this have been proposed including: over-transcription of certain genes (expected when a transcription repressor is decreased), abnormal gene repression, increased transcriptional noise, and downstream effects on other processes (Kerr and Ravine, 2003). Human point mutations have been reproduced in animals, and the degree of affinity of MeCP2 for methylated DNA correlates with severity of the mutation type for missense mutations. Although MeCP2 protein is still produced in missense mutations, an R106W mutation (which results in a severe human phenotype) decreases the affinity of MeCP2 for methylated DNA by 100-fold, whereas T158M (resulting in a less severe phenotype) only reduces binding moderately (Kudo et al., 2001). The least severe human phenotype associated with an R133C mutation in MECP2 displays similar DNA binding to that of the wild-type protein (Ballestar et al., 2000).

Both *MECP2* gain of function and loss of function cause severe neurodevelopmental disorders in humans. Although many phenotypic similarities to *MECP2* loss of function exist (intellectual disability, poor or absent speech, repetitive behaviors, seizures), individuals with *MECP2* duplication syndrome exhibit prominent anxiety, atypical social interaction, and recurrent infections (Ramocki et al., 2009; Van Esch, 2011). Based on animal studies, *MECP2* dosing has been correlated with both morphologic changes and dendritic spine density of neurons (Larimore et al., 2009). When rat embryonic hippocampal neurons are cultured with reduced levels of normal *MECP2*, shorter dendrites with normal axon length result, whereas mutant *MECP2* results in both shorter axons and dendrites. However, as one might hypothesize, overexpression by 2-fold of *MECP2* yields both longer axons and dendrites. In postnatal hippocampal slice cultures from the rat, decreased *MECP2* results in decreased spine density, while overexpression has no effect on spine density (Chapleau et al., 2009).

The excitatory-inhibitory balance is abnormal in Rett syndrome models, reflecting changes in multiple neurotransmitter systems (Shahbazian et al., 2002). In patients with Rett syndrome, CSF dopamine metabolites are reduced to 19% and serotonin metabolites to 23% of normal levels. This effect is more pronounced with severe mutations (Samaco et al., 2009). GABAergic neurons in the cortex express 50% more MeCP2 than other cortical neurons. When *MECP2* is knocked out in GABAergic cells, the human respiratory, compulsive, motor, and social phenotypes associated with Rett syndrome are recapitulated. In particular, repetitive behaviors that mimic human stereotypies are present (Chao et al., 2010). In astrocytes, dendritic and synaptic abnormalities have been associated with excessive glutamate secretion, but the clearance rate may be a culprit as well, as has been suggested by cultured knockout astrocytes with elevated glutamate clearance; this results in decreased down-regulation of excitatory amino acid transporters and excessive glutamate synthetase production (Okabe et al., 2012). Abnormal GABA release may explain prevalent seizures (Medrihan et al., 2008) while the motor and cardiorespiratory features seen in both humans and mouse models may be due to abnormal excitatory neurotransmitter release (Kron et al., 2012). When MeCP2 is selectively decreased in GABA-releasing neurons, the model exhibits repetitive behaviors, again similar to the human stereotypies, suggesting these may be due to abnormal GABAergic function (Chao et al., 2010).

The brainstem in Rett syndrome exhibits multiple abnormalities. One of these is abnormal serotonin transporter binding in the dorsal motor nucleus of the vagus, which may result in abnormal autonomic control and subsequent gastrointestinal and cardiac dysfunction (Paterson et al., 2005). In the hippocampus, synaptic connections are dysfunctional, and this could be associated with the deficits in socialization and motor apraxia in humans with Rett syndrome (Moretti et al., 2006). The hypothalamic-pituitary-adrenal axis also demonstrates abnormalities, including enhanced corticotropin-releasing hormone expression, and this could contribute to the anxiety which is prevalent in Rett syndrome (McGill et al., 2006). Brain-derived neurotrophic factor (BDNF) levels are lower than expected in the nucleus tractus solitarius, which may correlate with abnormal neuronal gating and cardiorespiratory abnormalities in Rett syndrome (Kline et al., 2010). Tyrosine hydroxylase expressing neurons are fewer in both the medulla and locus coeruleus, resulting in low levels of norepinephrine (Taneja et al., 2009). In human autopsy studies, patients with Rett syndrome have age-related changes in the glutamatergic system and NMDA receptors; at a younger age, NMDA receptor levels are increased, whereas, at an older age, NMDA receptor levels are decreased. These findings have been reproduced in *Mecp2* knockout mice (Blue et al., 2011) and may be explained by the potential regulation by MeCP2 of splicing of the NMDA subunit NR1 (Young et al., 2005). In support of this hypothesis, deletion of the NMDA receptor subunit NR2A prevents progressive visual loss in Mecp2 deficient mice (a feature not seen in humans with the disease, however; Durand et al., 2012). Collectively, these findings suggest two alternative models which remain unresolved. First, all these phenotypes are due to common MeCP2 gene targets that generate different phenotypic outcomes in different cell types or different brain regions. Alternatively, MeCP2 regulates gene expression in a cell and tissue-specific manner. These alternative hypotheses can be resolved by the identification of genes whose expression is regulated by MeCP2.

### If MeCP2 Regulates Gene Transcription, What Are Its Targets?

Remarkably, despite 20 years since the discovery that *MECP2* loss of function mutations cause Rett syndrome, only a handful of putative target genes have been identified, and both the degree to which MeCP2 regulates these and the direction of dysregulation remain unclear (Amir et al., 1999; Na et al., 2013). This is despite the clear picture of dysfunction present in multiple neurotransmitter systems. Techniques such as chromatin immunoprecipitation (ChIP) combined with RNA sequencing and/or quantitative proteomics, as we will discuss below, could solve this issue entirely. In fact, recent efforts pairing experimental design with mathematical modeling are heading in this direction (Cholewa-Waclaw et al., 2019).

Although one would expect mutations in a protein responsible for DNA methylation to result in derepression of genes, this is simply not the case—instead, modest increases and decreases in gene transcription are seen in tissues (Chahrour et al., 2008; Ben-Shachar et al., 2009). There are 1,200 neuronally expressed genes sensitive to MECP2 genetic defects, as demonstrated in mouse brain or human iPSC-derived neurons (Chahrour and Zoghbi, 2007; Chahrour et al., 2008; Tanaka et al., 2014). Few of these genes have been comprehensively analyzed.

Among the few examples, regulation of BDNF by Mecp2 is both important and paradoxical. The Mecp2 protein exhibits a repressive effect on the *Bdnf* promotor (Wade, 2004). One would predict that derepression of *Bdnf* in the *Mecp2* deficient animal would result in overexpression of the BDNF protein. However, in the knockout *Mecp2* mouse model BDNF levels are low (Sun and Wu, 2006). No satisfying explanation for this phenomenon exists, although researchers have hypothesized that either reduced synaptic activity on a global level or a feedback mechanism involving over-transcription of other repressors could decrease BDNF levels. If BDNF is overexpressed in the *Mecp2* knockout mouse, this results in partial rescue of the phenotype (Chang et al., 2006; Wang et al., 2006). One study found that Mecp2 regulates the squalene epoxidase gene in mice; this gene is critical for cholesterol metabolism, and the evidence from a large suppressor screen study in the mouse model is compelling for this association. These data were supported by a study of *MECP2* in cultured human fibroblasts (Buchovecky et al., 2013b; Segatto et al., 2014).

One strategy to sort out the targets of MeCP2 regulation involves biotin tagging in female mice expressing loss-of-function mutations that cause disease in humans (Johnson et al., 2017). Using this method, the authors identified a distinct difference in gene expression between wild type cells in these animals and cells harboring a disease-causing mutation. Furthermore, they identified differences in transcript expression between the mutations in fold-changes of the transcriptome. Unfortunately, this approach does not address the problem that decreased levels of MeCP2 could independently alter gene transcription, nor does it account for the poor correlation between transcriptome and proteome found in a number of studies (Gygi et al., 1999; Chen et al., 2002; Pascal et al., 2008; Ghazalpour et al., 2011; Yeung, 2011; Horvath and Monteggia, 2017). In terms of the general classes of genes found to be upregulated or downregulated, one study found that long genes are upregulated and another found the opposite to be true (Gabel et al., 2015; Johnson et al., 2017).

## Targeted Therapeutics—A Role for “Omics”?

Despite a paucity of mechanistic arrows to connect the dots between disease phenotypes and abnormal neurotransmitters and growth factors, a number of clinical trials have been undertaken to attempt to restore abnormalities in these systems. These clinical trials were conceived to attempt to rectify the downstream dysfunction identified in both human tissues and in animal models. We have published a detailed account of these studies, so will only briefly discuss them here (Katz et al., 2016). No current strategy for treating the underlying cause of Rett syndrome exists, i.e., restoring *MECP2* function. However, ten specific dysregulated systems have been identified which are amenable to currently available therapeutics. The burden of the disorder is so high that a number of clinical trials have been undertaken with varying degrees of preclinical evidence to support them. Each has held promise, and over half were conducted with a blinded, placebo-controlled design. Although all studies reported some positive or statistically significant results, and in many cases both physicians and caregivers believed the drugs were beneficial, none have led to the adoption of a clinically meaningful treatment beyond standard supportive care. In our detailed review of these studies, we discuss the possible reasons for what amounts to failed clinical trials. In some cases, the effect, if present, was trivial. In others, the effect appeared clear in specific individuals, but the overall effect on the group was negligible. In still other cases, the improvements described by physicians and caregivers were not adequately captured in the study outcome measures. The result in all cases was that the study results were difficult to interpret.

Clinical trials are both time consuming and expensive. In rare diseases, this point is driven home by the small potential participant pool, and the fatigue induced by asking the same families to participate in trial after trial. Moreover, recent proposed studies have included both more potent drugs, such as the dissociative anesthetic Ketamine, and more risky approaches, such as injectable drugs like Copaxone and Insulin-like Growth Factor-1. Most recently, treatment strategies have turned to gene therapy, approaches in which the wild type *MECP2* gene is added to neurons using a viral vector. However, uncertainty surrounds the gene therapy clinical trial planned for 2019, since *MECP2* dosing is critical and cannot be regulated by such an approach. The strategies behind “omics” have the potential to address all of these issues: first, by providing the earliest possible indication of cellular response, or target engagement; second, by monitoring response to the treatment, both for dosing and toxicity measurements; third, as a predictive biomarker to determine response of individual subjects; and fourth, as a surrogate biomarker for clinical response. Moreover, “omic” biomarkers could provide a window into monitoring in the clinic that would prove invaluable for anticipatory guidance and targeting resources like therapy services.

**Figure 1:**
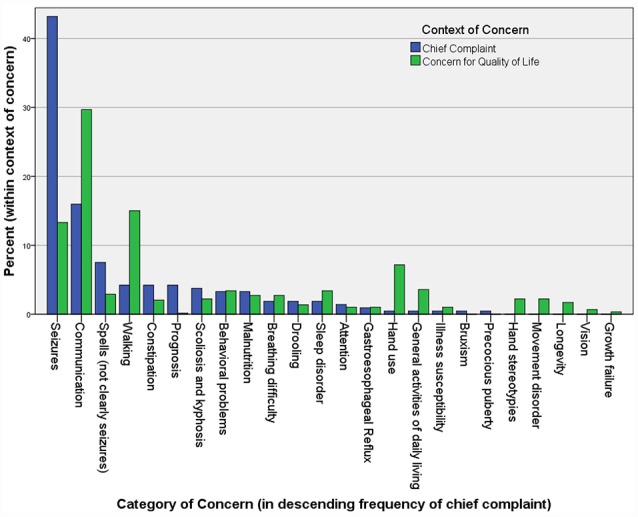
The chief complaints (221) and other “concerns” (586) in 113 Rett syndrome patients seen in the author’s (DT) clinic over a period of 2 years. Caregivers are asked to name their primary reason for the visit at the beginning of the interview. After discussing this and all related medical issues, they are asked to name the top three concerns they would address if they could. They are asked to name problems that are major contributors to morbidity that they would address if a “cure” were available, without considering available treatments. Unexpectedly, when posed this question at the end of a visit, caregivers often raise concerns for which a treatment actually exists, but that they failed to raise during the discussion of medical care.

One critical problem with therapeutic trials of drugs is that their efficacy evaluation mostly rests in clinical assessments. In Rett syndrome, the list of clinical concerns is long and complex, so summarizing these in the form of an outcome measure has proven difficult (Figure 1). Rett syndrome is heterogeneous on a number of levels; although four core criteria unite the group (loss of hand use and verbal language, hand stereotypies, and abnormal gait), the concerns of caregivers vary widely, and the factors contributing to disease burden are a moving target, often waxing and waning spontaneously.

A host of outcome measures and biomarkers have been used in clinical trials to try to capture this assortment of signs and symptoms (Table 1). Because none warrant the moniker “gold standard,” most trials have chosen an assortment of outcomes, rarely using the same metric more than once, all of which have amounted to exploratory measurements. We posit that systems biology and the use of comprehensive “omics” tools to identify biomarkers hold promise for not only detecting appropriate changes in functional gene product with treatment but also potentially providing a window to measure dosing of a vector-based treatment approach. The fundamental principle is that a complex system can be understood better by considering it in its entirety, including dimensions such as time, space, and context, rather than through naïve reductionism.

**Table 1:** Fifty-one outcome measures and biomarkers used in 25 clinical trials of Rett syndrome.

Category	Type	Metric
Outcome measure	Scale (Clinician)	Clinical Rett syndrome stage*
		Bayley scales of infant development
		Peabody Developmental Motor Scales
		Gesell developmental observation
		Mullen scales of early learning
		Motor behavioral assessment*
		Rett syndrome: SSI*
		Portage guide for early education (Gross/fine motor development, cognition, socialization)
		Hand Apraxia Scale
		Clinical Severity Score*
		Clinical global impression of severity and improvement
		Kerr Severity Scale*
		Aberrant behavior checklist
	Scale (Caregiver)	Vineland Adaptive Behavior Scales
		Patient well-being index
		Short-form 36
		Rett syndrome behavioral questionnaire*
		Anxiety, depression, and mood scale
		Visual analog scale of caregiver concerns
		Pediatric QOL (caregiver proxy)
		Screen for social interaction
	Clinical exam	Stereotypies
		Ambulation
		Bruxism
		Breathing dysregulation
		Social interaction
		Alertness
		Mobility and tone
		Behavior
		“Autonomic” function (breathing dysrhythmia, drooling)
		Visual attention
		Timed gait testing
	Caregiver diary	Percent reduction in seizures
		Seizure frequency
		Sleep patterns
		Overall improvement by parental report
Biomarker	Blood	Routine laboratory testing (complete blood count, serum chemistry)
		Brain-derived neurotrophic factor level
		Metabolic measures of oxidative stress
	Cerebrospinal fluid	Biogenic amines
		Brain-derived neurotrophic factor level
	Neurophysiology	Qualitative EEG features
		Quantitative EEG (frontal alpha asymmetry)
		EEG spike frequency
	Polygraphy	Induction plethysmography
		Polysomnography sleep efficiency
		Apnea index
		Respiratory disturbance index
	General physiology	Peripheral oxygen saturation
		Somatic growth
		Head circumference

Adapted from Katz et al. (2016). *Indicates Rett syndrome specific scale.

The process of global, unbiased querying of systems downstream of the genetic code, involving techniques referred to as “omics” or “multi-omics,” has opened the door to a vast amount of information about function, protein and genetic interactions, gene product expression, metabolite and lipid content, and complex feedback processes that integrate these molecules into pathways and in time and space. This approach has been called a “new era in *systems biology*.” We define systems biology as the study of “biological systems by systematically perturbing them (biologically, genetically, or chemically); monitoring the gene, protein, and informational pathway responses; integrating these data; and ultimately, formulating mathematical models that describe the structure of the system and its response to individual perturbations” (Ideker et al., 2001; Hood et al., 2004; Weston and Hood, 2004; Hillmer, 2015). Systems biology has the potential to connect the dots between dysregulation of a single protein and a complex phenotype like Rett syndrome (Hood et al., 2004; Weston and Hood, 2004; Haas et al., 2017). The components of the “omics” are described briefly below. Taken together each can be compared to the “phenome,” or the sum of traits exhibited by an organism and its component parts.

### Genomics

Studies the genome, which constitutes the complete genetic material of an organism. It contains the basal information for building organisms and their cells in their whole diversity. The ability to sequence the genome once held the promise of explaining all phenotypic characteristics of human disease. However, the sequence information in the genome is static and phenotypic outcomes in human disease emerge from interactions between the genome and environment.

### Epigenomics

Analyzes the modification of the structure of chromatin and modifications to DNA (such as methylation), which are referred to as the epigenome. The characterization of these modifications is the field of epigenomics. The epigenome is influenced by the environmental history of an organism, thus modifying gene expression and phenotypic outcomes. A number of known monogenic causes of autism and other neurodevelopmental disorders, including Rett syndrome, Fragile X syndrome, Angelman syndrome, and Prader-Willi syndrome, are caused by genes responsible for epigenetic modifications (Egger et al., 2004). As such, to understand the dysfunction wrought by mutations in these genes, we need to look downstream into gene expression.

### Transcriptomics

Measures the transcriptome, the set of all RNAs expressed by a cell, group of cells, tissue, or organ. The transcriptome provides information about when and where genes are activated or inactivated, therefore offering a proxy for the “functional” state of a cell, tissue, or organ. The entire transcriptome can be assessed using RNA-seq, which can yield information about the presence and expression levels of an RNA, as well as splice variants, gene fusion, mutations and modifications to RNAs occurring after their transcription such as editing (Wang et al., 2009; Spies and Ciaudo, 2015).

### Proteomics

Studies the proteome which, represents the entire set of proteins expressed by the genome of a cell, tissue, organ, or organism. The proteome bridges the gap between the genetic code and phenotypic expression. Proteomic complexity cannot be predicted fully from the transcriptome (see below), and is not completely understood using current technology (Harper and Bennett, 2016). Nonetheless, this approach has provided improved understanding of the pathophysiology of cancer, infectious diseases, pre-term birth, and common diseases such as hypertension (Romero et al., 2006; Casado-Vela et al., 2011; Waterer, 2012; Tebani et al., 2016; Jean Beltran et al., 2017; Arnett and Claas, 2018).

### Cistromics

The cistrome is the collection of all cis-acting targets associated to a particular trans-acting factor, such as MeCP2, at a genome-wide scale (Liu et al., 2011). Among the cistromic strategies, a powerful approach particularly relevant to *MEPC2* biology is ChIP. This technique is a hybrid of the previously mentioned strategies and permits identification of genome-wide DNA or RNA binding sites for transcription factors and other proteins. Sites are identified by immunoprecipitation of a desired protein with DNA or RNA binding capacity, followed by sequencing of the coprecipitated nucleic acid. This approach enables the identification of the putative binding sites of transcription factors, sites of epigenetic modifications in DNA and chromatin (ENCODE Project Consortium et al., 2007; ENCODE Project Consortium, 2011).

### Metabolomics, Lipidomics, and Ionomics

The interaction of products of the genetic code results in an assortment of measurable phenotypic characteristics, and these have been organized into the above categories, including metabolites, lipid components, and elemental components.

We argue that the use of each one of these omic approaches, alone or in combination, is uniquely poised to identify statistically prioritized mechanisms of disease and molecular biomarkers in neurodevelopmental disorders (Mullin et al., 2013). In the next section, we discuss Rett syndrome as a prime candidate to test the power of molecular systems biology and omics approaches in the discovery of mechanisms of disease and molecular biomarkers.

## Genotype-Phenotype Associations in Rett Syndrome: An Incomplete Story

Hundreds of specific *MECP2* mutations exist and the phenotypic variability of these is striking. Greater than 99% of these mutations are caused by mutations in the paternal germline, which are spontaneous; only the vast minority are inherited from mothers, who are carriers. A database cataloging both pathogenic and nonpathogenic mutations lists over 200 pathogenic mutations in *MECP2*, including eight common point mutations (four missense mutations and four nonsense mutations), and many 3′ truncations and deletions of entire exons. Together, these are found in more than 80% of individuals with Rett syndrome (Percy, 2011). In addition to the approximately 200 causative mutations, many mutations in *MECP2* have never been linked to neurodevelopmental disease (Krishnaraj et al., 2017). A minority have been associated with particularly mild cases, for example, the “preserved-speech” variant of Rett syndrome (Zappella, 1992; De Bona et al., 2000). Still others have been associated with altogether different syndromes. The A140V point mutation is the best example of this and causes PPM-X syndrome, consisting of psychosis, pyramidal signs, and macro-orchidism (Klauck et al., 2002). Although predominantly seen in males, an adolescent onset syndrome involving the A140V point mutation was described in a female with parkinsonian features and cognitive regression in adolescence (Venkateswaran et al., 2014). Although they demonstrate profoundly different human phenotypes, the mouse models that have been created with these specific human point mutations all exhibit the same neuropathological features, including abnormal neuropil density, and decreased dendritic complexity (Chapleau et al., 2009; Jentarra et al., 2010).

Considering the common mutations associated with the classic phenotype of the disorder, substantial clinical overlap exists, such that statistically significant differences in human phenotype among the mutations can only be found in large data sets between the absolute extremes of the genotypic severity scale (Cuddapah et al., 2014). In fact, it is not difficult to find an individual with the “mildest” mutation, R133C, who is phenotypically more severe than an individual with the most severe mutation, R168X. When specific components of the disease, such as seizure severity and breathing dysregulation are considered, although trends of severity can be found with respect to genotype, these are subtle, non-significant associations (Tarquinio et al., 2017, 2018).

Much of the clinical heterogeneity, even with identical point mutations, owes to the role of *MECP2* itself. The MeCP2 protein serves diverse functions that include modulation of DNA methylation, acetylation at lysine residues, interacting with RNA to influence splicing, and direct activation and repression of gene transcription. Because Rett syndrome is considered an X-linked dominant disease, lyonization (random silencing of one of the X-chromosomes in each cell early in embryonic development) has been invoked to explain this variability (Amir et al., 2000). Some individuals with very mild disease, and rare asymptomatic carriers have been identified and shown to have markedly skewed X-chromosome inactivation. Because testing can only be done easily on blood or buccal tissue, these tests only comment on peripheral silencing of the mutant gene. This is presumed to represent (to some unknown degree) X-chromosome inactivation in the brain (Huppke et al., 2006; Hardwick et al., 2007). Monozygotic twins are unusual but several pairs exist, and phenotypes are often different; this may be due to skewed X-chromosome inactivation (Ishii et al., 2001). However, X-chromosome inactivation does not explain most of the variability present in Rett syndrome (Bao et al., 2008), and may, in fact, be misleading (Takahashi et al., 2008). Other possible variables include clonal expansion of the mutant X-chromosome, but this is almost impossible to test clinically. The best example of these processes is the Calico cat, in whom patches of different hair color on every cat are the result of random distribution of X-chromosomes from the maternal and paternal cell lines during dermatogenesis. Because neurogenesis would exhibit similar clonal expansion, the distribution of mutant *MECP2* will randomly differ in various brain regions. This will occur even in Rett syndrome twins, even those with skewed X-chromosome inactivation. Although the distribution of mutant *MECP2* cannot be tested on neuronal tissue *in vivo* without invasive testing (Gibson et al., 2005), recent technological advances have made it possible to do so in select tissues (Renthal et al., 2018).

## Why Is Rett Syndrome an Ideal Neurodevelopmental Disorder to Tests Systems Biology to Identify Biomarkers?

Our quest for molecular biomarkers in Rett syndrome begins with the fundamental problem that there are no objective biological markers for diagnosing or evaluating any of the forms of autism spectrum disorder (Uddin et al., 2017). This fact is rooted in part on the complexity of the disease, with the majority of cases being polygenic, and the phenomenological diagnosis, which is defined by observational clinical features rather than standardized biochemical or molecular measurements (Bailey et al., 1996; Risch et al., 1999). Although no molecular biomarkers have been tied to *MECP2* dysfunction, Rett syndrome is one of the few monogenic forms of autism spectrum disorder (Katz et al., 2012; Leonard et al., 2017).

### Criteria for an Ideal Disorder to Test Molecular Biomarkers

Defining molecular biomarkers for autism spectrum disorder, or any neurodevelopmental disorder, could be best materialized by considering the following heuristic criteria:

1. Disorder definition should ideally be founded on unequivocal genetic diagnosis, as is the case with Rett syndrome, or any other monogenic neurodevelopmental disorder. Rett syndrome is caused by mutations in methyl-CpG-binding protein 2 (MECP2) in >95% of patients meeting consensus clinical diagnostic criteria (Neul et al., 2010, 2014; Cuddapah et al., 2014).
2. If the disorder is well-defined genetically, then the gene affected should ideally have loss- and gain-of-function mutations in humans with a certain degree of phenotypic overlap. MECP2 mutations are ideal in this regard, as Rett syndrome is the result of loss-of-function mutations in MECP2, while duplication of the MECP2 gene causes a distinctive syndrome, the MECP2 duplication syndrome, that shares autism symptoms with Rett (OMIM: 300005^3^; Ramocki et al., 2009; Lombardi et al., 2015; Leonard et al., 2017).
3. High phenotypic penetrance of the mutation and consistency should exist in the clinical phenotype. Rett syndrome manifests mostly with autism and intellectual disability symptoms (Percy, 2011). This is in contrast with other neurodevelopmental disorders that can present themselves as multiple psychopathologies, even though the genetic defects are well defined, as is the case with copy number variations (Girirajan et al., 2011; Rutkowski et al., 2017).
4. There should be some knowledge about mechanisms of disease at any biological complexity level. Mechanisms of disease exist in a pathogenesis continuum along increasing levels of biological complexity. This continuum spans from the mechanisms most proximal to the mutation, such as is the role of *MECP2* as a transcriptional regulator, to mesoscale processes affected by the mutation, like cell and tissue mechanisms, to macroscale phenotypes at the level of circuit or anatomical brain dysfunction.
5. Animal and cellular models of disease should genetically and phenotypically reproduce disease features (Katz et al., 2012). These animal models are essential because they offer unlimited experimental access to all tissues, developmental stages, and levels of biological complexity along a pathogenesis continuum.
6. Cell and tissue analysis should not be constrained to neurons and brain tissue, even if the most salient pathology and clinical features point to the brain. This assertion is founded on the observation that most brain genes are expressed in diverse tissues (Uhlén et al., 2015). We would like to emphasize that in addition to searching for common mechanisms of disease shared among tissues, the advantage of conceptualizing disease as a systemic/multiorgan disorder is the immediate translational implication that biomarkers of disease could be explored in accessible tissues. For example, we could sample biomarkers in patient tissues, such as muscle, or fluids more accessible than the brain. Take for example genes involved in lipid and cholesterol metabolism, whose expression is controlled by MECP2 in brain cortex and liver (Buchovecky et al., 2013a; Kyle et al., 2016). The concept that organ-specific diseases express molecular phenotypes in multiple tissues other than the affected organ has been tested comprehensively in mouse models of organ-specific pathologies (Kozawa et al., 2018).

Rett syndrome fulfills some of these criteria for the search of biomarkers. However, we still know little about mesoscale cell and tissue mechanisms disrupted by MECP2 genetic defects (Katz et al., 2012). Despite this, we have a plethora of information about the most mutation-proximal mechanisms of MECP2 loss-of-function as a transcriptional regulator and the circuit consequences of MECP2 mutations (Na et al., 2013). The most proximal mechanisms to the mutation stem from the molecular function of MECP2 as a transcriptional regulator/repressor capable of inducing up- or down-regulation of gene transcription (Lyst and Bird, 2015; Cholewa-Waclaw et al., 2018). Nearly 1,200 neuronally expressed genes are sensitive to MECP2 genetic defects, as demonstrated in mouse brain or human iPSC-derived neurons (Chahrour and Zoghbi, 2007; Chahrour et al., 2008; Tanaka et al., 2014). These transcripts are involved in processes including neuronal differentiation, neuronal morphology and size, and function of excitatory and inhibitory synapses (Smrt et al., 2007; Chahrour et al., 2008; Na et al., 2012; Qiu et al., 2012; Yang et al., 2012). These facts about the diversity of MECP2 transcriptional targets raise key questions related to the identification of Rett syndrome molecular biomarkers: First, do gene expression products sensitive to MECP2 expression converge on discrete pathways that can be scrutinized? If there exists a molecular pathogenesis, is it shared among different cell types, regions, and developmental stages of the brain? Finally, are MEPC2 molecular mechanisms associated with MECP2-deficiency in the brain shared by non-neuronal tissues? These critical questions should inform where, when, and how we search for molecular biomarkers of disease. However, the answers to these questions still await resolution.

We favor cellular, tissue, and organ mesoscale gene and protein expression analyses of proteins or RNAs to identify potential biomarkers in animal cells and tissues as a first step. These findings can then be translated to human samples. Expression analyses allow facile exploration of biomarkers while considering the challenges and questions just described. Results from cell to organ mesoscale searches can be scaled down to be interpreted and tested in the context of mechanistic hypotheses closer to the role of MECP2 in transcriptional regulation. Conversely, the disruption of these biomarkers can be assessed in macroscale mechanisms of disease to assess their contribution to circuit dysfunction or anatomical phenotypes. The most comprehensive approach to identify mesoscale mechanisms of disease and potential biomarkers is the genome-wide interrogation of gene expression. As described above, expression can be measured at the level of coding and non-coding regulatory RNAs, as well as the proteins, transcriptomes and proteomes, respectively. Transcriptomes sample expression across the whole genome of a cell, tissue, organ, or biological fluid. The proteome coverage is at a half of all encoded proteins in humans, which are estimated to be around 20,000 (International Human Genome Sequencing Consortium, 2004; Beck et al., 2011; ENCODE Project Consortium, 2011; Nagaraj et al., 2011; Wilhelm et al., 2014). Proteomes and transcriptomes have the added advantage of being hereditable molecular phenotypes, allowing their use in family trait studies (Wu et al., 2013; Parts et al., 2014; Wright et al., 2014; Huang et al., 2015). In the case of cellular proteomes, we have demonstrated they follow genealogical relationships among subjects within a pedigree and segregate those with the disease from their non-diseased/unaffected family members (Gokhale et al., 2018; Zlatic et al., 2018). This strategy can be carried further with the pairing of classical twin studies, a number of which have been published in Rett families, and the novel techniques discussed here (van Dongen et al., 2012). The proteome has the distinctive advantage of being the executor of phenotypic programs in cells and tissues. Thus, it has the highest probability of identifying biomarkers of disease and disease mechanisms not yet recognized.

Expression levels between proteomes and transcriptomes partially correlate in normal tissues and cells (Maier et al., 2009; Ghazalpour et al., 2011; Vogel and Marcotte, 2012). This is in part due to interplay between the coding transcriptome and the non-coding transcriptome that modulates the extent of protein expression. The partial correlation between coding transcriptome and proteome is likely to be disrupted in Rett syndrome. Defects in MECP2 alter the expression of regulatory non-coding RNA that in turn influences translation of defined mRNAs (Klein et al., 2007; Im et al., 2010; Cheng et al., 2014; Tsujimura et al., 2015). Surprisingly, even though we have catalogs of genes whose RNA expression is regulated by MECP2, we have limited understanding at a global scale of how MECP2-dependent transcriptome modifications translate into protein expression profiles in MECP2 deficient cells and tissues. Only one recent study compares the transcriptome and proteome of symptomatic Mecp2 null male mice, yet the authors report global expression correlations (Pacheco et al., 2017). In general, other proteome studies in Rett syndrome are limited in number, rely on outdated technology, and are of small sample size (Matarazzo and Ronnett, 2004; Cortelazzo et al., 2013, 2014, 2017).

The present status of “omic” technologies and the power of bioinformatic tools to distill information out of complex datasets calls for their use in renewed studies on monogenic and polygenic forms of neurodevelopmental disorders, in particular, Rett syndrome. Importantly, proteomes and transcriptomes could catalyze the discovery of cell-to-organ mesoscale disease mechanisms and biomarkers in Rett syndrome. This discovery potential stems from the capacity of these technologies to comprehensively and unbiasedly sample molecular phenotypes, irrespective of how distal a molecular phenotype is from its genetic defect.

## The Relevance of Deeper Understanding of Mecp2 Function

Caregivers of individuals with Rett syndrome recognize the degree of dysfunction on many levels. Although the diagnostic criteria consist of four components, the concerns raised by caregivers evoke a more complete picture of both the neurological and systemic implications of the disorder (Figure 1). One can envision a monitoring biomarker that could be used to gauge dysfunction in specific pathways downstream of MeCP2. This could be used to titrate drugs used commonly in Rett syndrome at present, but currently introduced in a trial-and-error fashion. Caregivers cite their concern about prognosis, and, although rare, sudden death does occur in Rett syndrome. A prognostic biomarker could help identify individuals who are at risk, and more careful monitoring could be prescribed, whereas those at low risk could be safely reassured. We hope that this suite of omics biomarkers will some day be useful in clinical trials as a tool to determine target engagement or even a reasonably likely surrogate endpoint. Although we face a long road before such an approach may be validated, the cost of *not* pursuing this course is high. Families are already burdened by the clinical trials they are being asked to participate in at present, in terms of time, emotional, and financial costs. We owe it to them to provide metrics of improvement in measures that we can have confidence are reliable markers of improvement, and could lead to clinically meaningful change.

### Hope for the Future

While our understanding of how mutations in *MECP2* cause the Rett syndrome phenotype remains incomplete, one important question has, in part, been answered. Researchers seeking to determine if a path to a clinically meaningful treatment is possible asked whether or not mature animals with defective Mecp2 could benefit from administration of the normal protein? Administering the protein and transferring it to the nucleus of neurons is technically difficult, but one elegant experiment engineered *Mecp2* null animals with a transgene. This allowed *Mecp2* to only be expressed in post-mitotic neurons. Because these animals were essentially identical to wild type animals, they concluded that *Mecp2* in postmitotic neurons could possibly rescue the phenotype in null animals. Subsequently, a genetic “switch” to silence Mecp2 in mice was engineered that could be activated after the mouse phenotype was evident. Once these mice were symptomatic, their native *Mecp2* was reactivated, and this restored a majority of function in the animals. Although this cannot be currently executed in humans, these experiments serve as proof of principle that both systemic and neurological defects, both phenotypic and those in synaptic plasticity, could be potentially reversed in mature animals if normal Mecp2 were present in the cell nuclei (Guy et al., 2007). In these and subsequent experiments, function is restored more robustly when Mecp2 is reactivated earlier in life, but rescue of the phenotype even occurs in adult mice (Robinson et al., 2012).

Although this review focuses on Rett syndrome, a number of studies of animal models of diseases including Down syndrome, neurofibromatosis type 1, tuberous sclerosis, Rubinstein–Taybi syndrome, fragile X syndrome and Angelman syndrome all suggest that neurodevelopmental deficits could be reversed, even in adult mice (Gadalla et al., 2011). We are hopeful that resolving the mesoscale gap and illustrating how cells, tissues and organs behave in the presence of a *MECP2* mutation using omics can provide a path to clinically meaningful change for these children over the next decade. Studies involving gene therapy are currently in various stages of development, both in Rett syndrome and other disorders, and these could result in profound clinical improvement over the next decade. Understanding the entire picture of how *MECP2* mutation results in the clinical phenotype of Rett syndrome through omics will allow us to design and test molecular biomarkers for response to these gene therapy strategies, and may allow the development of personalized medicine strategies to aid in the successful completion of clinical trials involving gene therapy.

## Data Availability

All datasets generated for this study are included in the manuscript.

## Author Contributions

DT and VF participated in study conceptualization, conduct of review, data collection, manuscript preparation, and approved the final manuscript as submitted. MW and AC participated in manuscript preparation, and approved the final manuscript as submitted.

## Conflict of Interest Statement

The authors declare that the research was conducted in the absence of any commercial or financial relationships that could be construed as a potential conflict of interest.

## Footnotes

^1^https://reverserett.org/research/consortia/outcome-measures-and-biomarkers-development/

^2^https://www.sfari.org/resource/sfari-gene/

^3^https://www.omim.org/entry/300005?search=mecp2&highlight=mecp2
